# Analysis of Hydrometallurgical Methods for Obtaining Vanadium Concentrates from the Waste by Chemical Production of Vanadium Pentoxide

**DOI:** 10.3390/ma15030938

**Published:** 2022-01-26

**Authors:** Ulyana Kologrieva, Anton Volkov, Irina Krasnyanskaya, Pavel Stulov, Dmitry Wainstein

**Affiliations:** 1 State Scientific Centre, I. P. Bardin Central Research Institute of Ferrous Metallurgy, 23/9 bdg. 2, Radio Street, 105005 Moscow, Russia; rhenium@list.ru (A.V.); iakrsn@gmail.com (I.K.); pavel1411@rambler.ru (P.S.); d_wainstein@sprg.ru (D.W.); 2 Surface Phenomena Researches Group, Staropimenovskiy Lane, 6, bdg.1, app. 4, 127006 Moscow, Russia

**Keywords:** vanadium, vanadium pentoxide, concentrate, waste, sludge, leaching, reduction, vanadium alloys, precipitation, hydrolysis

## Abstract

The paper describes hydrometallurgical methods to recycle wastes of vanadium pentoxide chemical fabrication. Sludges containing a significant amount of V_2_O_5_ can be considered as an additional source of raw materials for vanadium production. We studied the one-stage leaching method using various iron-based reductants for converting V^5+^ to V^4+^ in a solution allowing to precipitate V when its concentration in the solution is low. As a result of the reduction leaching with further precipitation, we obtained concentrates with V_2_O_5_ content of 22–26% and a high amount of harmful impurities. Multistage counterflow leaching can be used to fabricate solutions with vanadium pentoxide concentration suitable for vanadium precipitation by hydrolysis and adding ammonium salts. The solutions with V_2_O_5_ content of ≈15 g/L can be obtained from the initial sludge by three-stage counterflow vanadium leaching. A concentrate with a content of 78 wt% V_2_O_5_ can be precipitated from these solutions at pH = 2.4 by adding ammonium chloride. Additionally, concentrate with V_2_O_5_ content of ≈94 wt% was precipitated from the solution with a concentration of >20 g/L V_2_O_5_ obtained from the roasted sludge. The concentrates were purified for increasing the vanadium content to 5–7%. The consumption and technological parameters of the considered processes are presented in the paper.

## 1. Introduction

Vanadium is an important strategic metal widely used in various fields of industry. In ferrous metallurgy, V in the form of ferrovanadium is used for steel alloying [1,2,3] and in non-ferrous metallurgy in aluminum-vanadium alloys for alloying titanium-based structural materials used in aerospace engineering (engines, fuselages of high-speed aircraft) [4,5,6,7]. In the chemical industry, vanadium compounds are used as catalysts [8]. Additionally, materials based on vanadium oxides (V_2_O_3_, VO_2_ and V_2_O_5_) are used in memristors (resistors with memory), bolometers (thermal infrared detectors), biosensors [9,10]. The application of vanadium in medicine for the manufacture of dental implants is described in [11].

Currently, the main sources for vanadium production are titanomagnetite ores [12,13,14,15]. Additionally, vanadium is extracted from coal [16,17,18], fly ash [19,20], spent catalysts [21,22], and other sources [23]. Titanomagnetite ores are processed to produce vanadium converter slag, from which vanadium pentoxide is obtained by chemical means [7,12,24,25,26,27,28].

Vanadium in converter slags is presented mainly in spinel (FeO·V_2_O_3_) practically insoluble in acid solutions. Therefore, in order to convert vanadium into soluble forms, preliminary slag oxidation roasting is carried out [7,12,24,25,26,27,28,29,30,31,32,33].

During the alkaline roasting, the formation of toxic salts of hexavalent chromium CrO_4_^2−^ [7,12,32] is possible. With this technology due to the complexity of disposal, exhausted sodium-containing solutions are thrown into the water basin polluting the environment. Roasting of slag with calcium-containing additives (lime, limestone) is considered more environmentally friendly when vanadium in the spinel is forming acid-soluble phases CaV_2_O_6_, Ca_2_V_2_O_7_ and Ca_3_V_2_O_8_ [7,12,33].

Vanadium is precipitated from leaching solutions by hydrolysis, as a result, technical vanadium pentoxide with impurities Mn, Si, Fe, etc., is acquired. To obtain a cleaner vanadium pentoxide, precipitation is carried out with the addition of ammonium salts (for example, NH_4_SO_4_, NH_4_Cl) [7,12,30]. Thus, the concentration of solutions before precipitation is usually higher than 15 g/L V_2_O_5_.

A significant amount of vanadium remains after leaching (<4.5 wt% V_2_O_5_) [33,34,35,36] in sludges (wastes of hydrometallurgical production of vanadium pentoxide), therefore, it can be considered as a technogenic source of raw materials for vanadium production. Vanadium in sludges similarly to initial vanadium slags exists in the form of spinel (V^3+^), also part of vanadium is in acid-soluble forms V^4+^ and V^5+^ [36]. Consequently, hydrometallurgical methods that are used in the production and research practice of vanadium slags processing should also be effective for this type of vanadium-containing raw materials.

The research was aimed at studies of hydrometallurgical methods of vanadium-containing sludge processing to produce vanadium concentrates.

Previously [34,35] it was shown that multi-stage counterflow leaching is necessary to obtain vanadium concentrates from sludges suitable for further smelting of vanadium alloys. In three stages of leaching, solutions with V_2_O_5_ content of ≈10 g/L can be obtained from the initial sludges and more than 20 g/L V_2_O_5_ from the roasted sludges in two stages.

It is known that vanadium can exist in aqueous solutions in the form of compounds with oxidation degrees from +2 to +5 [37,38]. All known methods of vanadium precipitation are based on the vanadium extraction into a concentrate in the form V^5+^. In this work, new methods for vanadium precipitation in the form of V^4+^ using reagents that are not used in production practice are studied. Additionally, the production of vanadium concentrates by ammonium methods was studied.

## 2. Materials and Methods

### 2.1. Materials

The original vanadium-containing sludges were obtained from the EVRAZ Vanadii Tula plant (Tula, Russia). The samples were obtained at various periods and the content of V_2_O_5_ somewhat differs.

The chemical analysis was performed with the X-ray fluorescence spectrometer AXIOSmax Advanced (PANalytical, Almelo, The Netherlands) using the method described in [36,39]. Table 1 shows the chemical composition of sludges.

### 2.2. Methods

#### 2.2.1. Leaching

The important parameters of the leaching process are the following: concentration of the sulfuric acid solution, leaching temperature and duration, and solid to liquid ratio S/L. To select optimal process parameters the samples were leached by H_2_SO_4_ solution with a concentration of 1–20% at 20–80 °C during 5–60 min and S/L = 1/1–1/10 (g/mL).

The leaching process was performed in the 10-L stainless steel reactor with an upper agitator. After the end of leaching, the pulp was filtered under vacuum and washed with water at a ratio of S/L = 1/0.5 (g/mL). The filtrate and washing water were not mixed.

Chemical analysis of leaching solutions before and after vanadium deposition was carried out using an atomic emission spectrometer with inductively coupled plasma Agilent 725 Radial (Agilent Technologies, Santa Clara, CA, USA). Standard solutions from High-Purity Standards were used for calibration.

Leaching rate was calculated as:

η(V_2_O_5_)_sol_ = m(V_2_O_5sol_)/m(V_2_O_5total_) × 100, % (1)

where V_2_O_5sol_—the mass of V_2_O_5_ in solutions after leaching, g; V_2_O_5total_—the total mass of V_2_O_5_ in the sample of sludge, g.

#### 2.2.2. Reducing Leaching

Reducing leaching was carried out by two methods using iron sulfate and metallic iron powder as reducing agents.

In the first method, FeSO_4_·7H_2_O (99 wt% FeSO_4_·7H_2_O) was added to the solution after sulfuric acid leaching of the sludge under optimal conditions and pulp filtration. The filtrate was heated to 80 °C using a heating plate with constant stirring, then brucite Mg(OH)_2_ was added to increase pH = 2–3 and FeSO_4_·7H_2_O was added at the rate of 1 g per 1 g of V_2_O_5_ in solution. After that, the pH of the solution was adjusted to ≈5.5 by adding brucite and the solution was kept under constant heating and stirring for 1 h.

In the second method, metallic iron powder (99.7 wt% Fe) was added during the process of vanadium leaching from sludge. Water and concentrated H_2_SO_4_ dropwise to pH ≈ 1.6 were added to the sample of sludge (at optimal S/L), the pulp was heated to 80 °C. Metallic iron powder was added at the rate of 2 g per 1 g of V_2_O_5_. Stirring and heating were carried out for 1 h. The resulting solution after filtration was heated to 80 °C once more, Mg(OH)_2_ was added to pH ≈ 5.5 and exposed 1 h.

After filtration and washing the sediment was roasted in a muffle furnace at 550 °C for 2 h, then the content of the main components in the vanadium-containing concentrate was measured.

The chemical composition of brucite, wt% was the following: moisture 0.20; calcination loss 32.0; MgO 64.5; Al_2_O_3_ 0.06; SiO_2_ 1.00; CaO 2.15; Fe_2_O_3_ 0.15.

Recovery rate of vanadium into the concentrate from the sludge was calculated as:

η(V_2_O_5_)_conc_ = m(V_2_O_5conc_)/m(V_2_O_5total_) × 100, % (2)

where V_2_O_5conc_—the mass of V_2_O_5_ in the concentrate, g; V_2_O_5total_—the total mass of V_2_O_5_ in the sample of sludge, g.

#### 2.2.3. Multistage Counterflow Leaching

To obtain a strong V_2_O_5_ solution from the original and roasted sludge (>10 g/L V_2_O_5_), multi-stage counterflow leaching was carried out. This method includes the following: the filtrate after leaching with H_2_SO_4_ solution in the first stage is used for leaching a new portion of the charge in the second stage; filtrate after the second stage is used similarly for the third stage, and so on until the final solution reaches a concentration V_2_O_5_ ≈ 10 g/L.

#### 2.2.4. V_2_O_5_ Sedimentation by Ammonium Salt

V_2_O_5_ was sedimented from solutions with a content of V_2_O_5_ > 10 g/L by adding solid ammonium chloride NH_4_Cl (99.5 wt% NH_4_Cl) at a flow rate of 30–45 g/L and pH = 1–8. For this, the solution was preliminarily neutralized by adding NaOH (99 wt% NaOH) in the form of granules permanently monitoring the pH values using a pH-meter.

The recovery rate of V_2_O_5_ into the concentrate from the solution was calculated as Equation (2), substituting the value of V_2_O_5_ content in the solution into the denominator.

## 3. Results and Discussion

### 3.1. Selection of Leaching Conditions

**Figure 1:**
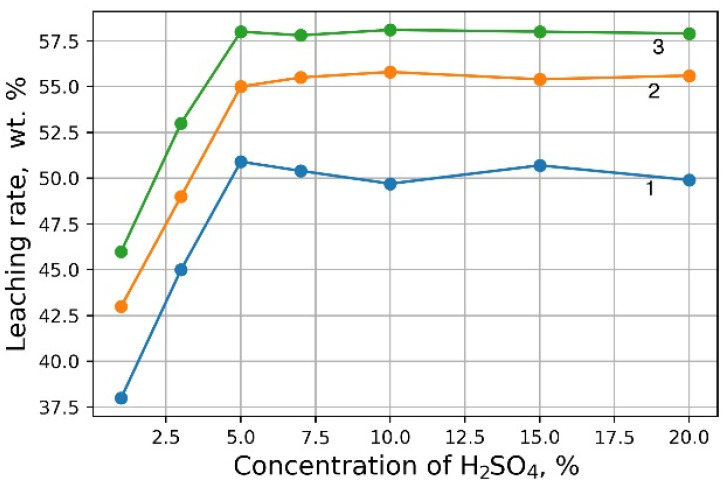
Effects of H_2_SO_4_ concentration on the recovery rate of V_2_O_5_ into solution at leaching temperature 20 °C (1), 60 °C (2) and 80 °C (3).

**Figure 2:**
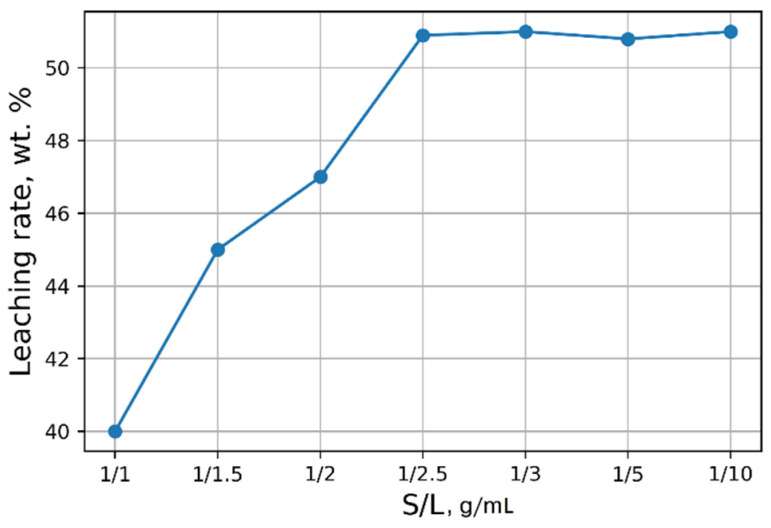
Effects of S/L on the recovery rate of V_2_O_5_ into solution.

**Figure 3:**
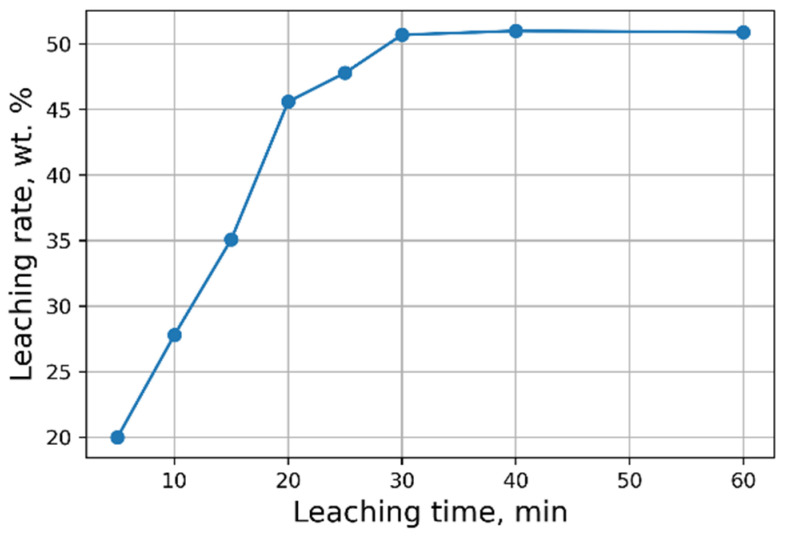
Effects of leaching time on the recovery rate of V_2_O_5_ into solution.

Investigations aimed at the selection of optimal leaching conditions were carried out on a sample of sludge No. 3 (Table 1). Figure 1 demonstrates the outcomes of H_2_SO_4_ concentration on the recovery rate of V_2_O_5_ into solution from the initial sludge at different temperatures and S/L = 1/5 (g/mL). The maximal recovery rate of V_2_O_5_ into solution (58%) is achieved at the leaching temperature of 80 °C and concentration of 5% of H_2_SO_4_ solution, without heating, the recovery rate of V_2_O_5_ is ≈50%. The leaching process at the temperature of 80 °C has such a disadvantage, as the need for constant monitoring of the level S/L due to significant evaporation of the leaching solution. As can be seen from Figure 1, heating has a small effect on the recovery rate of V_2_O_5_, thus it was decided to conduct the experiments without heating at 5% H_2_SO_4_ solution, while the concentration of V_2_O_5_ in the solution was ≈3 g/L. The optimal S/L ratio was found to be 1/2.5 (g/mL) (Figure 2). The maximum concentration of vanadium in the solution is reached 30 min after the start of the process (Figure 3).

The optimal conditions for two-stage counterflow leaching of roasted sludge with 1 wt% CaCO_3_ additive were selected in [33]: H_2_SO_4_ concentration of the solution is 5–7%, the leaching time is 20 min at each stage of the process; S/L = 1/2.5. These data were used in this work for obtaining vanadium-concentrated solutions with its further precipitation by brucite and NH_4_Cl.

### 3.2. Reducing Leaching

For studies of vanadium leaching with its further reduction by FeSO_4_·7H_2_O, sludges with V_2_O_5_ content of 2.25 wt% were used (Table 1, sample No. 1).

**Figure 4:**
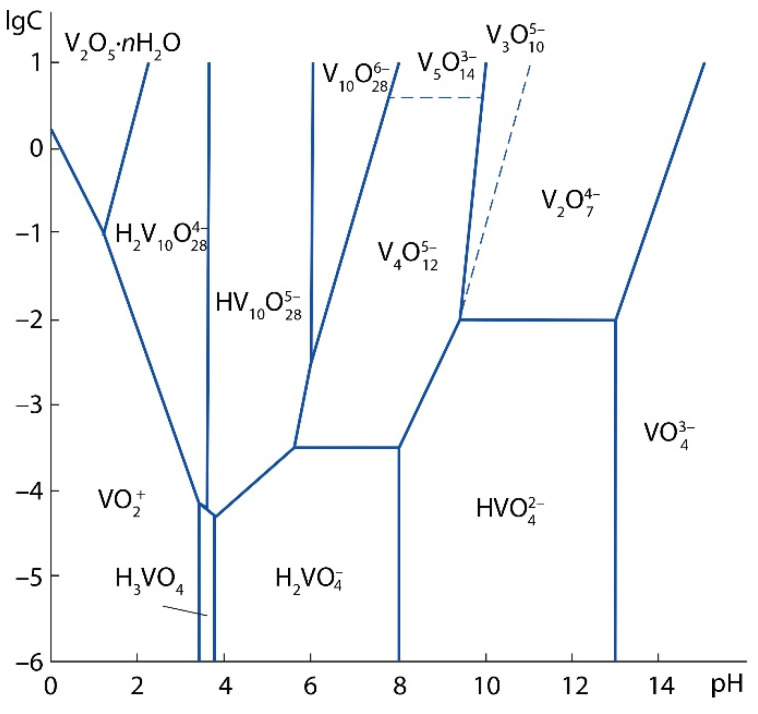
Diagram of the state of oxo- and hydroxoforms of vanadium (V^5+^) in aqueous solutions.

The test sample contains 0.94 wt% V_2_O_5a.s._, i.e., this amount of V_2_O_5_ can be converted into a solution by leaching with a 7% H_2_SO_4_ solution [33]. Vanadium can be represented in the acid-soluble part in the form of salts: orthovanadates (Me_3_VO_4_), pyrovanadates (Me_4_V_2_O_7_) and metavanadates (MeVO_3_), where Me is a monovalent metal ion. Since the sludge under study is a product of vanadium converter slag processing roasted with limestone, the most possible acid-soluble phases in the sludge are Ca_3_(VO_4_)_2_, Ca_2_V_2_O_7_, Ca(VO_3_)_2_. As a result of the treatment of calcium vanadates with a solution of sulfuric acid, various vanadium ions can be formed depending on the pH of the solution and the concentration of vanadium, (Figure 4) [38].

When vanadium is leached with a 5% H_2_SO_4_ solution, at which the pH of the resulting solution was 0.6–0.8, and the vanadium concentration in the solution after leaching was ≈ 3 g/L, the formation of VO_2_^+^ ions is probable by the following reaction:

VO_4_^3−^ + 4H^+^ → VO_2_^+^ + 2H_2_O. (3)

For example, the reaction could occur as followed:

Ca_3_(VO_4_)_2_ + 4H_2_SO_4_ → 3CaSO_4_ + (VO_2_)_2_SO_4_ + 4H_2_O (4)

In acidic medium at pH = 2–3 VO_2_^+^ is reduced to VO^2+^ by iron sulphate with the following reactions:

VO_2_^+^ + 2H^+^ + ē → VO^2+^ + H_2_O, (5)

Fe^2+^ − ē → Fe^3+^. (6)

The leaching solution acquired a blue color after the addition of FeSO_4_·7H_2_O crystals indicating the predominance of V^4+^ in solution.

**Figure 5:**
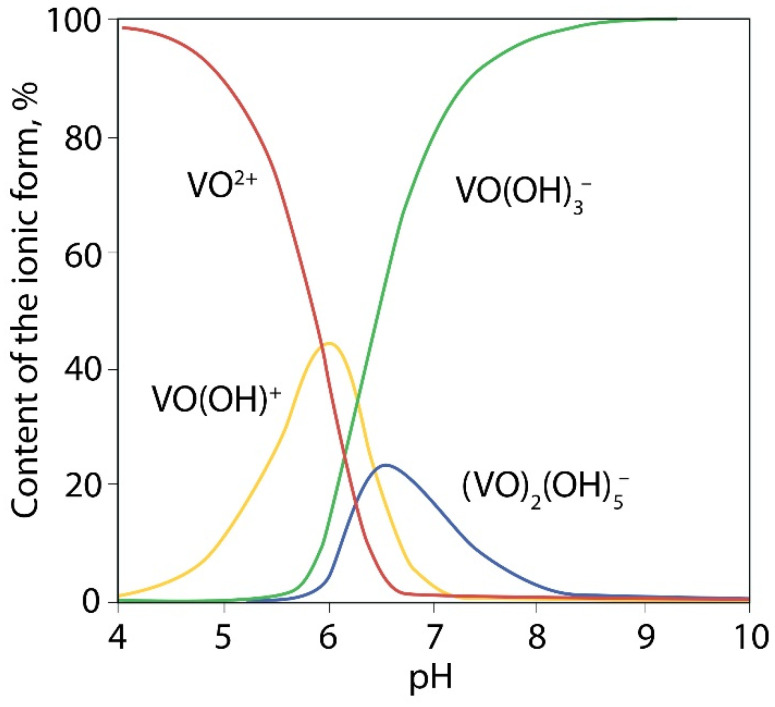
Distribution diagram effect of pH on of the forms of V^4+^ in 10 nM solution.

It is known that VO^2+^ ions in aqueous solutions are existing mainly in the form of [VO(H_2_O)_5_]^2+^ when pH < 3.5 and as [VO(OH)]^+^ at higher pH values (Figure 5) [40]. At pH > 4 precipitation of VO(OH)_2_ occurs by reaction:

VO^2+^ + 2OH^−^ = VO(OH)_2_↓ (7)

After precipitation by brucite and roasting of the residuum, a light brown concentrate was obtained. As one can see in Table 2, it was possible to obtain a concentrate with V_2_O_5_ content of ~22%. This concentrate contains a significant content of impurities, including phosphorus which is a harmful impurity for ferrous metallurgy. Thus, this concentrate requires further processing before obtaining vanadium alloys.

Investigations on reduction by metallic iron were carried out using sludge with V_2_O_5_ content of 2.78 wt%. (Table 1, sample No. 2). Optimizing the process, we added iron for vanadium reduction during the leaching process and heated the pulp to 80 °C immediately after iron powder addition. As the concentrate obtained by reduction with iron sulfate contains a significant amount of phosphorus, the pH value was increased to ≈ 1.6. Reduction of V^5+^ passing into solution by metallic iron can proceed by the following reactions:

(VO_2_)_2_SO_4_ + Fe + 2H_2_SO_4_ → 2VOSO_4_ + FeSO_4_ + 2H_2_O (8)

2VOSO_4_ + Fe + 2H_2_SO_4_ → V_2_(SO_4_)_3_ + FeSO_4_ + 2H_2_O (9)

In this process, solutions with V_2_O_5_ content of ≈6.1 g/L were obtained, while the recovery rate of V_2_O_5_ into solution was 36.5 wt%. The final concentrate after roasting in the muffle furnace contained 26.5 wt% of V_2_O_5_. Thus, metallic iron as a reducing agent allowed to increase V_2_O_5_ content slightly and to reduce phosphorus content in the final concentrate (see Table 2).

Methods of one-stage reduction leaching with followed precipitation by brucite are ineffective for processing sludge due to the low vanadium content and high content of harmful impurities in the resulting concentrates as shown by investigations.

The method of reducing leaching with of FeSO_4_·7H_2_O additive was investigated for the recovery of vanadium from solutions with a concentration of ~20 g/L obtained under optimal conditions from roasted sludge with 1% CaCO_3_ additive by two-stage counterflow leaching. The process was carried out as previous studies with FeSO_4_·7H_2_O additive, and as a result, a concentrate with content V_2_O_5_ of 53.6 wt% was obtained (Table 3). This concentrate is characterized by a significant content of impurities, however, due to the high vanadium content, it may be suitable for smelting vanadium ligatures [40].

Table 4 presents the technological parameters and consumption parameters of the considered processes of reducing leaching and subsequent precipitation in terms of 1 g of the resulting concentrate.

### 3.3. V_2_O_5_ Precipitation by Ammonium Salt

Investigations of vanadium concentrate production by precipitation with ammonium salt NH_4_Cl from solutions with a content of V_2_O_5_ > 10 g/L were carried out using initial sludge (Table 1, Sample No. 3) and roasted sludge with 1% CaCO_3_ additive.

A final solution with a concentration of 15 g/L V_2_O_5_ and pH = 0.65 was obtained from the initial sludge by three-stage counterflow leaching with 5% H_2_SO_4_ solution at the first stage. It is known that vanadium precipitates from an acidic solution at pH = 1.8–3 and at pH = 4–8 [12]. At pH = 1.8–3, vanadium can precipitate in the form of ammonium hexavanadate by reaction:

3V_10_O_28_^6−^ + 10NH_4_^+^ + 8H^+^ = 5(NH_4_)_2_V_6_O_16_ + 4H_2_O (10)

As a result of reaction with NH_4_Cl ammonium metavanadate is formed from alkaline solutions:

VO_3_^−^ + NH_4_^+^ → NH_4_VO_3_ (11)

**Figure 6:**
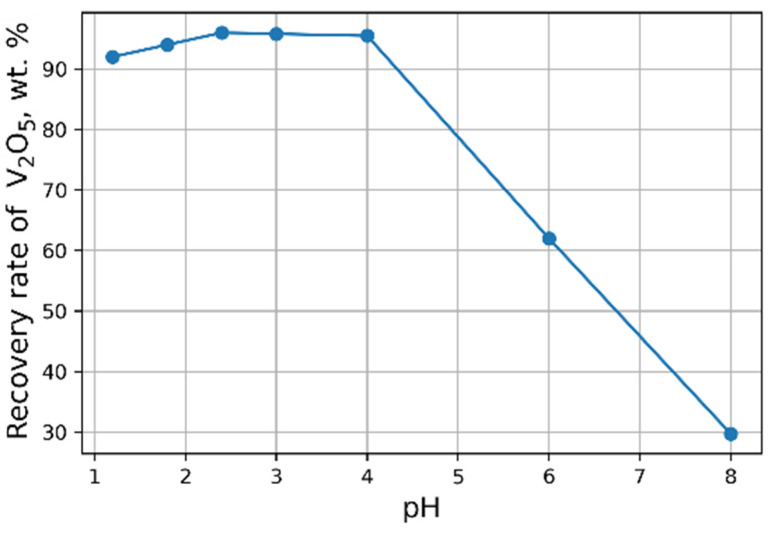
Effect of pH value on the recovery of V_2_O_5_ into the concentrate (30 g/L NH_4_Cl flow rate).

**Figure 7:**
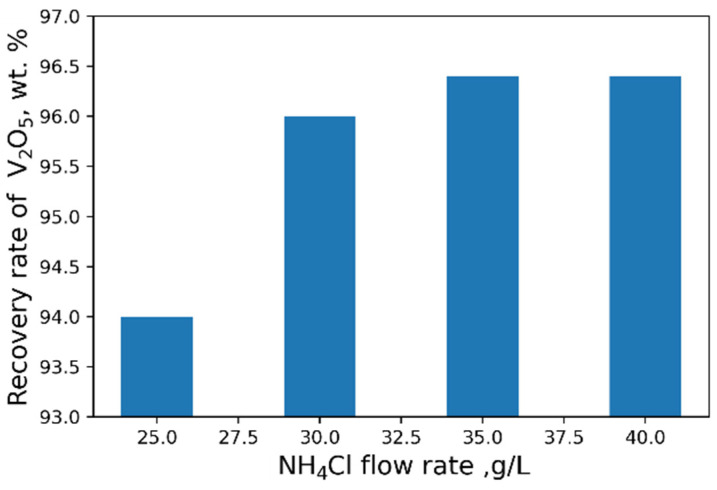
Effect of NH_4_Cl flow rate on the recovery of V_2_O_5_ into the concentrate.

The influence of pH on the recovery rate of V_2_O_5_ into the concentrate is shown in Figure 6. Tests at pH < 3 were carried out heating solutions to 95 °C. The maximal recovery rate of 96 % of V_2_O_5_ into the concentrate from the solution is achieved at pH = 2.4. An increase in NH_4_Cl flow rate does not practically affect the yield of V_2_O_5_ (Figure 7).

Compositions of concentrates at different pH values are presented in Table 5. With an increasing pH, the recovery rate of V_2_O_5_ into the concentrate decreases, and an increasing amount of impurities is also observed.

Let us consider the possibility of V_2_O_5_ precipitation from a solution with a concentration of V_2_O_5_ 23 g/L by ammonium salt obtained after two-stage counterflow leaching of roasted sludge with 1% CaCO_3_ additive.

**Figure 8:**
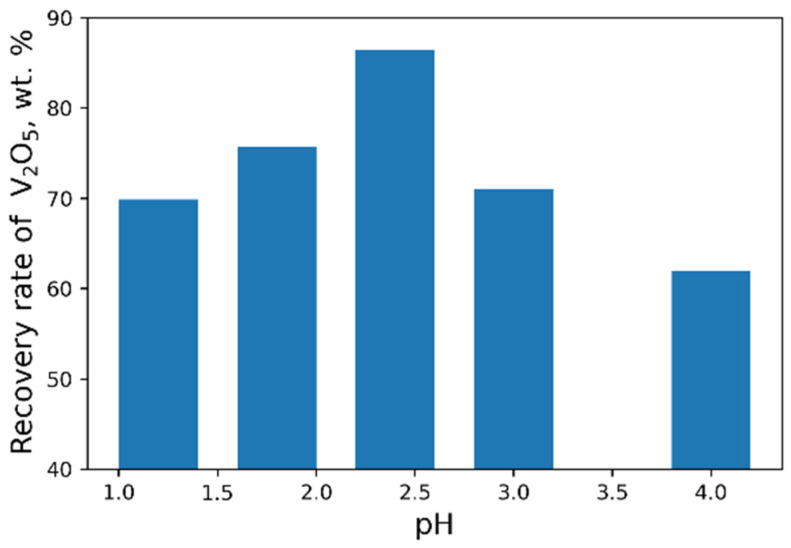
Effect of pH value on the recovery of V_2_O_5_ into the concentrate (45 g/L NH_4_Cl flow rate).

**Figure 9:**
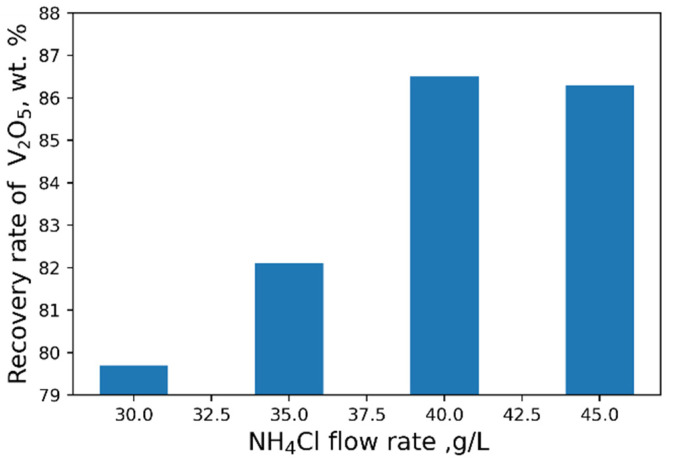
Effect of NH_4_Cl flow rate on the recovery of V_2_O_5_ into the concentrate.

As a result of our studies, the following optimal parameters were selected: pH = 2.4, 40 g/L NH_4_Cl flow rate (Figure 8 and Figure 9). At optimal conditions, we obtained the concentrate with V_2_O_5_ content of 93.6 wt% (Table 6). However, higher purity vanadium pentoxide requires additional stages of concentrate processing.

Reducing the content of impurities in concentrates obtained by precipitation from solutions with V_2_O_5_ concentration of 15 and 23 g/L at pH = 2.4 (Table 5 and Table 6) was conducted on their washing by repulpation. Washing was carried out with 1% NH_4_Cl solution at S/L = 1/10 at a temperature of 95 °C. In this process the removal of soluble sulfates of manganese, iron, titanium, and alkali metals takes place. Due to the low solubility of vanadium compounds in ammonium chloride solution, the losses of V_2_O_5_ into washing solutions were less than 0.5%. Washing of the concentrate obtained from the initial sludge allowed to increase V_2_O_5_ content to 84.94 wt% (Table 7), which is slightly lower than the requirements of the standard (≥90 wt%). Additionally, the concentrate has an increased phosphorus content unsuitable for smelting high-vanadium alloys, such as FeV60, FeV80. The concentrate obtained from the roasted sludge meeting to the grade of VNO-2 (Table 7). To obtain a cleaner vanadium pentoxide (pure, chemically pure), additional stages of washing from impurities will be required. The parameters of the considered processes are presented in Table 8.

Parameters of the analyzed processes for obtaining vanadium concentrates from sludges are presented in Table 9. The most effective technology includes preliminary oxidation roasting of the sludge, two-stage leaching of vanadium from the roasted sludge with a sulfuric acid solution and its further precipitation by hydrolysis or ammonium salts.

## 4. Conclusions

Waste from the production of vanadium pentoxide is undoubtedly a promising technogenic source of raw materials for vanadium production. The experimental analysis of various sulfuric acid methods of sludge processing has shown that the most effective method is an oxidation roasting of sludge with further two-stage counterflow leaching of vanadium with 5% H_2_SO_4_ solution in the first stage at S/L = 1/2.5 (g/mL). Precipitation from solutions with a high concentration of more than 20 g/L by hydrolysis or ammonium salts allows fabricating concentrates with >90 wt% V_2_O_5_ satisfying the requirements of the standard.

Technologies of counterflow leaching of vanadium from the initial sludges can also be used to obtain vanadium concentrates, but in this case, V_2_O_5_ content is significantly lower: 72–78 wt% V_2_O_5_$ V_2_O_5_ content increases after additional purification to 85 wt%, at the same time a significant amount of vanadium remains in the waste sludge. In ferrous metallurgy, such concentrates can be used for the low vanadium ferroalloys (FeV40) and ligatures production.

Methods of reducing leaching with subsequent precipitation of vanadium with brucite have not proven their effectiveness due to the high content of impurities in obtaining concentrates.

## Figures and Tables

**Table 1:** Chemical composition of vanadium-containing sludge samples, wt%.

Component	Sludge No.		
	#1	#2	#3
V_2_O_5_	2.25	2.78	3.59
V_2_O_5a.s_	0.94	1.61	1.4
MgO	1.36	1.53	1.04
Al_2_O_3_	2.28	2.2	1.56
SiO_2_	11.69	11.2	11.8
P_2_O_5_	0.03	0.03	0.03
K_2_O	0.10	0.11	0.11
CaO	12.2	11.4	12.1
TiO_2_	7.14	7.69	7.3
Cr_2_O_3_	3.13	3.14	3.34
MnO	6.50	6.67	6.52
Fe_2_O_3_	40.9	39	36.8
SO_3_	14.5	14.25	15.81

V_2_O_5a.s_—acid-soluble form of V_2_O_5_. Method of determination is described in [33].

**Table 2:** Chemical composition of initial sludges, products and wastes of reducing processes.

Component	Content (wt%) with Using Reducing Agent					
	FeSO_4_·7H_2_O			Metallic Iron		
	Sludge Initial	Waste Sludge	Concentrate	Sludge Initial	Waste Sludge	Concentrate
V_2_O_5_	2.25	1.55	22.1	2.78	1.41	26.5
V_2_O_5a.s._	0.94	0.1	n/d *	1.61	0.30	n/d
Mn_total_	6.50	4.54	1.82	5.20	4.33	4.58
Fe_total_	40.9	28.4	9.3	27.3	30.1	15.7
SiO_2_	11.69	10.6	5.19	11.2	11.0	5.52
Al_2_O_3_	2.28	2.12	4.23	2.20	2.14	4.51
TiO_2_	7.14	7.48	0.77	7.69	7.59	0.17
K_2_O	0.1	0.081	n/d	0.11	n/d	n/d
MgO	1.36	2.34	36.2	1.53	5.7	27.1
CaO	12.2	10.76	5.50	11.4	8.56	1.7
P_2_O_5_	0.03	0.005	0.52	0.03	0.014	0.16
S_total_	5.8	6.12	5.02	5.70	6.0	2.43
Cr_2_O_3_	3.13	3.24	n/d	3.14	n/d	0.25

* Not defined.

**Table 3:** Chemical composition of roasted sludge, product and waste of reducing process from solution with concentration V_2_O_5_ of ~20 g/L.

Component	Content, wt%		
	Roasting Sludge with 1 % CaCO_3_ Additive	Waste Sludge	Concentrate
V_2_O_5_	3.55	0.61	53.6
V_2_O_5a.s._	3.50	n/d	n/d
Al_2_O_3_	1.54	1.91	0.64
CaO	12.79	13.3	4.11
Fe_2_O_3_	36.43	38.0	18.3
K_2_O	0.11	n/d	n/d
MgO	1.03	1.42	13.3
MnO	6.45	4.72	4.84
P_2_O_5_	0.03	n/d	0.45
SO_3_	15.84	17.44	0.54
SiO_2_	11.5	12.1	3.96
TiO_2_	7.23	6.91	0.26

**Table 4:** Parameters of reducing leaching.

Parameter	Value at Using a Reducing Agent during Leaching		
	One-Stage		Multistage
	FeSO_4_∙7H_2_O	Fe Metallic	FeSO_4_∙7H_2_O
*Leaching*			
Content V_2_O_5_ in solution, g/L	3.05	6.1	20.0
Solution pH	0.5	1.6	0.70
Fe flow rate, g/g concentrate	–	0.023	–
*Precipitation*			
Mg(OH)_2_ flow rate up to pH ≈ 3, g/g concentrate	1.33	–	1.20
FeSO_4_∙7H_2_O, g/g concentrate	0.22	–	0.55
Mg(OH)_2_ flow rate to pH ≈ 5.5, g/g concentrate	0.74	0.15	0.80
Content V_2_O_5_ after precipitation, g/L	0.2	0.45	1.97
*Concentrate*			
Concentrate yield, g/kg sludge	42.3	29.2	27.4
Recovery rate of V_2_O_5_ into the concentrate, wt. %	29.3	33.4	56.0

**Table 5:** Chemical composition of initial sludge and concentrates after precipitation at different pH values.

Component	Content, wt%			
	Sludge Initial	Concentrate (pH = 2.4)	Concentrate (pH = 4)	Concentrate (pH = 8)
V_2_O_5_	3.59	78.0	60.4	45.0
V_2_O_5a.s._	1.4	n/d	n/d	n/d
Al_2_O_3_	1.56	0.81	1.03	1.34
CaO	12.1	0.07	0.09	2.43
Cr_2_O_3_	3.34	n/d	n/d	n/d
Fe_2_O_3_	36.8	16.92	14.92	9.01
K_2_O	0.11	n/d	0.08	0.03
MgO	1.04	0.13	0.12	8.21
MnO	6.52	0.35	1.33	16.2
Na_2_O	n/d	0.32	10.9	7.07
P_2_O_5_	0.03	0.88	0.9	0.54
SO_3_	15.81	0.25	8.08	6.67
SiO_2_	11.8	0.38	0.59	1.14
TiO_2_	7.3	1.89	1.3	1.03
Cl	0.0	n/d	0.26	1.33

**Table 6:** Chemical composition of initial sludge and concentrate obtained after V_2_O_5_ precipitation by NH_4_Cl.

Component	Content, wt%	
	Roasting Sludge with 1 % CaCO_3_ Additive	Concentrate (pH = 2.4)
V_2_O_5_	3.55	93.6
V_2_O_5a.s_	3.50	n/d
Al_2_O_3_	1.54	1.05
CaO	12.79	0.03
Fe_2_O_3_	36.43	2.42
K_2_O	0.11	n/d
MgO	1.03	n/d
MnO	6.45	0.2
Na_2_O	n/d	0.96
P_2_O_5_	0.03	0.5
SO_3_	15.84	0.8
SiO_2_	11.68	0.17
TiO_2_	7.23	0.21
Cl	0.0	0.06

**Table 7:** Chemical composition of washing concentrates obtained from initial and roasted sludges by counterflow leaching method.

Component	Content (wt%) of Washing Concentrates, Obtained from Sludges	
	Initial	Roasted
V_2_O_5_	84.94	97.12
Al_2_O_3_	0.5	0.7
CaO	0.05	0.03
Fe_2_O_3_	11.2	1.7
MgO	0.09	n/d
MnO	0.1	0.1
Na_2_O	0.5	0.3
P_2_O_5_	0.5	0.1
SO_3_	0.18	0.2
SiO_2_	0.38	0.17
TiO_2_	1.56	0.18
Cl	n/d	0.1

**Table 8:** Parameters of counterflow leaching method.

Parameter	Sludge	
	Initial	Roasted
*Leaching*		
Content V_2_O_5_ in solution, g/L	15	23
Solution pH	0.65	0.70
*Precipitation*		
NaOH flow rate дo pH = 2.4, g/L	60	60
NH_4_Cl flow rate, g/L	35	40
Content V_2_O_5_ after precipitation, g/L	0.8	3.1
*Washing of Concentrates*		
NH_4_Cl flow rate, g/g concentrate	0.1	0.1
*Concentrate after Washing*		
Concentrate yield, g/kg sludge	14	21
Recovery rate of V_2_O_5_ into the concentrate, wt%	36.6	52

**Table 9:** Comparative table of parameters of various hydrometallurgical processes of sludge processing.

Method	Material	Leaching Parameters	V_2_O_5_ Precipitation	Concentrate	
				V_2_O_5_ Content_,_ wt%	Recovery Rate V_2_O_5_ from Sludge_,_ wt%
Counterflow leaching	Initial sludge (3.08 wt% V_2_O_5_) [34]	3 stages; 5% H_2_SO_4_ solution at 1 stage; S/L = 1/2.5 (g/mL)	Hydrolysis, 95–98 °C; pH = 1.4; 1 h	71.2	35.0
	Roasted sludge (3.75 wt% V_2_O_5_) [35]	2 stages; 5% H_2_SO_4_ solution at 1 stage; S/L = 1/2.5 (g/mL)	Hydrolysis, 95–98 °C, pH = 1.0; 1 h	90	45
	Initial sludge (3.59 wt% V_2_O_5_)	3 stages; S/L = 1/2. (g/mL)5; 5% H_2_SO_4_ solution at 1 stage	NH_4_Cl 35 g/L; additional washing 1% NH_4_Cl solution	85	36.6
	Roasted sludge (3.55 wt% V_2_O_5_)	2 stages; S/L = 1/2.5 (g/mL); 5% H_2_SO_4_ solution at 1 stage	NH_4_Cl 40 g/L; additional washing 1% NH_4_Cl solution	97	52
Reducing leaching	Initial sludge (2.25 wt% V_2_O_5_)	S/L = 1/2.5 (g/mL); 5% H_2_SO_4_ solution; reducing agent FeSO_4_·7H_2_O	Mg(OH)_2_; pH = 5.5; 1 h	22.1	29.3
	Initial sludge (2.78 wt% V_2_O_5_)	S/L = 1/2.5; t = 80 °C; pH = 1.6; Fe metallic reducing agent	Mg(OH)_2_, pH = 5.5; 1 h	26.5	33.4
Counterflow leaching + Reducing leaching	Roasted sludge (3.55 wt% V_2_O_5_)	2 stages; 5% H_2_SO_4_ solution at 1 stage; S/L = 1/2.5 (g/mL); reducing agent FeSO_4_·7H_2_O	Mg(OH)_2_; pH = 5.5; 1 h	53.6	56

## Data Availability

Data are contained within the article.
